# Virtual reality to improve low-back pain and pelvic pain during pregnancy: a pilot RCT for a multicenter randomized controlled trial

**DOI:** 10.3389/fmed.2023.1206799

**Published:** 2023-09-04

**Authors:** Francisco-José García-López, José-Manuel Pastora-Bernal, Noelia Moreno-Morales, María-José Estebanez-Pérez, Antonio Liñán-González, Rocío Martín-Valero

**Affiliations:** ^1^Department of Physiotherapy, Faculty of Health Science, University of Malaga, Malaga, Spain; ^2^Department of Physiotherapy, Faculty of Health Science, Campus of Melilla, University of Granada, Melilla, Spain; ^3^Department of Nursing, Faculty of Health Science, Campus of Melilla, University of Granada, Melilla, Spain

**Keywords:** virtual reality, physiotherapy, low-back pain, pelvic pain, pregnancy

## Abstract

A significant proportion of women experience low back and pelvic pain during and after pregnancy, which can negatively impact their daily lives. Various factors are attributed to these complaints, and many affected women do not receive adequate healthcare. However, there is evidence to support the use of different physiotherapeutic interventions to alleviate these conditions. Virtual reality is a promising complementary treatment to physiotherapy, particularly in improving pain perception and avoidance. The primary objective of this study is to evaluate the efficacy of a four-week program combining VR and physiotherapy compared to standard physiotherapy in pregnant women with low back and pelvic pain, in terms of improving pain avoidance, intensity, disability, and functional level. The study also aims to investigate patient satisfaction with the VR intervention. This research will be conducted through a multi-center randomized controlled clinical trial involving pregnant patients residing in the provinces of Seville and Malaga with a diagnosis of low back and pelvic pain during pregnancy. The alternative hypothesis is that the implementation of a Virtual Reality program in combination with standard physiotherapy will result in better clinical outcomes compared to the current standard intervention, which could lead to the development of new policies and interventions for these pathologies and their consequences.

**Clinical trial registration**: clinicaltrials.gov, identifier NCT05571358.

## 1. Introduction

Roughly 50% of women experience low back pain (LBP) during pregnancy, and approximately 25% still suffer from pain 1 year after giving birth (1). LBP and pelvic pain (PP) are common complaints during pregnancy, which may worsen as pregnancy progresses and in some cases may even radiate to the buttocks, legs, and feet (2). The reported global prevalence rates for these conditions vary widely, ranging from 24 to 90%, mainly due to the lack of a universally accepted disease classification system (3). For many women, the pain can become severe enough to interfere with daily activities, disrupt sleep, and have negative impacts on social and sexual life, work capacity, and psychological well-being and contributes to high levels of sick leave (4). There are various reasons that can be associated with back pain during pregnancy. One of the factors is mechanical stress due to the growing uterus, resulting in lumbar lordosis (5). Additionally, the separation of abdominal muscles during pregnancy can also cause strain on the paraspinal muscles. The hormone relaxin, which is increased during pregnancy, is also a contributing factor, as it leads to joint laxity and instability, which can cause rotational movements in the sacroiliac joints. These factors have been identified as possible causes of back pain during pregnancy (6, 7).

According to estimates, more than half of women receive insufficient or no healthcare intervention for conditions such as LBP and PP (8). Guidelines in Europe recommend managing LBP and PP by providing patients with sufficient information and a sense of security are necessary to enable individuals to carry out their daily tasks without disruption, staying active and working where possible, and offering tailored exercises as needed. Prenatal healthcare providers in the United Kingdom and Nordic countries typically educate pregnant women about effective ways to handle lower back pain, pelvic pain, or both during pregnancy and may suggest they seek physiotherapy for targeted treatment (9). In contrast, women in the United States are frequently informed that experiencing lower back pain during pregnancy is a normal occurrence. To alleviate such pain, a range of approaches have been implemented, including exercise, rest, hot and cold compresses, support belts, massage, acupuncture, chiropractic care, aromatherapy, relaxation techniques, herbal remedies, yoga, Reiki, paracetamol, and nonsteroidal anti-inflammatory drugs (3, 4, 8, 9). Other therapies have also been studied, such as exercise, yoga, manual therapy, acupuncture, and multi-modal approaches. A 2015 Cochrane systematic review and meta-analysis found that regular exercise has been shown to potentially lower pregnancy-related LBP, enhance functional ability, and decrease the need for sick leave compared to usual prenatal care (2). A 2018 systematic review of 32 studies concluded that prenatal exercise can reduce the severity of LBP and PP during and after pregnancy compared to not exercising (1).

Some studies addressed the issue of sick leave during pregnancy, presenting positive results through exercise programs, reducing healthcare costs and promoting women’s health (10, 11). For persistent LBP lasting more than 12 weeks, recommended physical treatments include an activity or exercise program that is gradually increased in intensity and aimed at enhancing functionality and preventing additional disability. Current evidence does not support the superiority of any particular type of exercise for managing pregnancy-related lower back pain, and therefore, guidelines suggest customizing exercise programs based on individual needs, preferences, and abilities. While some guidelines do not recommend passive therapies such as spinal manipulation or mobilization, massage, and acupuncture, others consider them optional and may recommend a brief course of treatment for individuals who do not respond to other interventions (12). For individuals with persistent lower back pain that has not responded to previous treatments, other passive therapies like ultrasound, transcutaneous electrical nerve stimulation, progressive relaxation, mindfulness-based stress reduction, and combined physical and psychological treatments may be options to consider (13–15). In cases where patients have not responded to initial treatments and are significantly functionally impaired by pain, multidisciplinary rehabilitation programs may be more effective than standard treatments. These programs typically include supervised exercise therapy, cognitive-behavioral therapy, and medication to help manage pain and improve function (13–18).

A clinical practice guidelines in LBP during pregnancy in Spain suggests the use of aquatic exercises and other individualized exercise programs, as well as therapeutic massage to relieve LBP during pregnancy (19). In addition, strengthening the muscles of the lumbo-sacral joint and pelvic girdle through physiotherapy has been shown to effectively alleviate back pain (20). Incorporating exercise as a treatment option for pregnant women with back pain aims to reduce their pain levels and mitigate associated health complications. This approach also seeks to enhance their overall quality of life (6).

Virtual Reality: In the last 20 years, virtual reality (VR) technology has advanced rapidly and is now widely used (21). VR refers to computer simulations that utilize interaction devices and sensory display systems (22, 23). This technology has been applied to various fields, including healthcare, where it has been used to provide treatment, aid in pain management, and support rehabilitation programs (12, 24, 25), among other clinical applications.

A systematic review from 2019 conclude that VR has the potential to improve outcomes for spinal pain with demonstrated statistical and clinical significance (26). Additional patient populations VR interventions may be particulary beneficial for individuals who are experiencing higher levels of pain, and physical dysfunction, as well as anxiety, an alternative treatment to opioid analgesics (26). A study conducted on 80 female breast cancer patients at a specialized cancer center in Jordan revealed that VRi can be an effective intervention for managing pain and anxiety. The study found that using immersive VR in conjunction with other interventions is more effective than using morphine alone for relieving pain and anxiety (27). In stroke patients VR show promise as a future tool in the rehabilitation of daily live activities, particularly in the subacute phase (27).

VR enables users to engage with computer-generated environments and simulate real-life exercises and situations. In the context of rehabilitation, motivation is a crucial factor that affects the outcome of a patient’s performance (26). By providing enriched environments with multiple sensory feedbacks (auditory, visual, tactile) and moving avatars, VR stimulates various neural circuits that enhance a patient’s learning and recovery process (28–30). Therefore, VR has the potential to aid patients in improving their movements and perception of body position and reducing pain during the VR exercises (31). In turn, VR is a tool with a powerful contextual factor with the capacity to modify the patient’s context, that is, it can modify dysfunctional expectations and beliefs to improve musculoskeletal pain. Mainly they find it useful with violation strategies when our patients have a negative expectation with prior with low presicion. On the other hand, besides VR, other tools also used are exercise and manual therapy. All these tools used appropriately are very useful for the modification of expectations and beliefs (32). We know that contextual factors can trigger positive or negative effects on the achievement of goals, therefore attending to these factors can improve daily clinical practice (33).

A review suggests that VR may be a tool capable of modifying patients’ body perception. That is, VR has the ability to explicitly or implicitly modify the body and spatial perception of patients with musculoskeletal pain (34). Thus it can be presented as a very interesting tool on a perceptual level (32, 33). This supports and relates it to the modification of the patient’s expectations and perception.

A systematic review from 2019 focus on orthopedic rehabilitation conclude that the promising evidence suggests that VR can be effective in treating chronic neck pain and shoulder impingement syndrome. In cases of rheumatoid arthritis, knee arthritis, ankle instability, and post-anterior cruciate reconstruction, VR and exercise have similar effects. However, the evidence regarding the effectiveness of VR in comparison to exercise in cases of fibromyalgia and knee arthroplasty is either inconclusive or absent (35). A recent systematic review conducted in 2020 indicates that VR exercises have the potential to produce positive physiological, psychological, and rehabilitative outcomes in individuals when compared to traditional exercise (36). VR technology can also be utilized for a variety of purposes during different stages of pregnancy such as reducing anxiety levels, training individuals to manage pain during labor effectively (37), lowering anxiety levels before cesarean, episiotomy repair, dilation, and curettage (38–41), reducing pain (28), and managing exercise training (24). The importance of external focus in exercise management was picked up in the review by Piccoli et al. (42). VR makes it possible to administer exercise by shifting the patient’s attention with musculoeskeletal disorder to the objetive of the task facilitating motor performance and learning. This implies that VR is a useful tool for managing externally focused exercises (42).

In addition to all these positive effects, it is important to note that VR has adverse effects such as motion sickness (MS). MS is a pathology that can cause various signs and symptoms such as nausea, vomiting, disorientation, sweating, fatigue, and headache. Currently, MS is being studied in the context of two main technologies, automated cars and VR, and is a pathology to be taken into account as it represents a threat to the success of therapy and acceptance (43).

Although VR has shown effectiveness in treating some orthopedic conditions, currently, there is no conclusive evidence available on the effectiveness of interventions utilizing VR in treating LBP and PP during pregnancy. Therefore, it is advisable to conduct further studies to evaluate the effectiveness of VR interventions in this population both in hospital environments and other areas of care, considering the current health scenario. The primary aim of this study is to assess the efficacy of a combined VR and Physiotherapy 4-weeks program compared to a standard physiotherapy intervention in LBP and PP in pregnant women to improve pain-related fear avoidance, pain intensity, disability and functional level. As secondary aim is to investigate patient satisfaction with the VR intervention.

## 2. Materials and methods

### 2.1. Design

This research is a 4-week prospective multicentre randomized clinical trial. Participant recruitment and the supervised VR program component will be provided by clinical setting at department of Physiotherapy at University of Sevilla and Málaga (Spain). This study encompasses various departments of gynecology rehabilitation, physiotherapy, and researchers from the University of Granada, University of Málaga, and University of Sevilla. All participants in this study will be treated in academic centers and facilities, in both cases belonging to the universities of Seville and Malaga city. The study adheres to the Standards for Quality Improvement and Excellence in Reporting (SQUIRE) guidelines (44) and is conducted in accordance with (CONSORT) Consolidated Standards of Reporting Trials criteria (45). In addition, it is based on Standard Protocol Items: Recommendations for Interventional Trials (SPIRIT) 2013 explanation and elaboration: guidance for protocols of clinical trial (46). More information in Supplementary Material.

The study was approved by the institutional ethics committee of Andalucía with internal code 1928-N-21. It has also been registered in the clinicaltrials.gov database under the trial registration number NCT05571358. All female participants must provide informed consent prior to enrollment in the study (Supplementary Material).

### 2.2. Participants and eligibility criteria

The trial will enroll pregnant women who report or have been clinically diagnosed with LBP, PP, or a combination of both.

To be eligible, patients must reside in Sevilla or Málaga during the intervention phase, and must not have had a history of LBP or lumbar pathology prior to pregnancy, or have experienced LBP or PP events before their first contact with the research team. Patients with absolute or relative contraindications such as heart disease, chronic obstructive lung disease, diabetes mellitus, incompetent cervix/cerclage, multiple gestation, risk of premature labor, preeclampsia/pregnancy-induced hypertension, thrombophlebitis, pulmonary embolism, intrauterine growth restriction, or serious blood disease, history of abortion or curettage will be excluded. Additionally, excluding patients who lack the cognitive ability to utilize modern technological tools will be necessary. The inclusion and exclusion criteria can be found in Table 1. The trial involves the participation of gynecology rehabilitation and physiotherapy departments, as well as researchers from the University of Granada, University of Málaga, and University of Sevilla.

**Table 1:** Inclusion and exclusion criteria.

Inclusion criteria	Exclusion criteria
Adult woman over 18 years old	Patients who had LBP or PP prior to their pregnancy.
Pregnant women experiencing symptomatic LBP, PP, or both conditions (47)	Patients who do not possess the cognitive ability required for the use of technological tools
Pregnant women in their second or third trimester, between the 12th and 38th week of gestation (2)	Patients with either absolute or relative contraindications.
Pain intensity rated as greater than 4 out of 10 on the VAS, indicating moderate to severe pain (47)	
Participants must reside in either Sevilla or Málaga during the research period	

LBP, Low Back Pain; PP, Pelvic Pain; VAS, Visual Analogy Scale.

### 2.3. Recruitment

To gather sufficient data for the development of this study, a sample size of 66 patients (*n* = 66) will be enlisted. To date, no studies have reported on the use of VR and LBP in pregnant in low resource setting; so that this randomized, blinded clinical trial will provide evidence for the effect size. However, an online sample size calculator was used^1^ to determine minimal sample size (accessed on 26 July 2023). Included in the calculation was a one-tailed test, we assumed a medium effect size of 0.65 based on related study on a similar topic (1, 47–50), a significance level of 0.05 and power of 0.8. As the first estimate of effect size, a sample size of 66 participants has been calculated, with an expected proportion of losses (10%), and a proportional distribution for each arm of the study (EG = 30 and CG = 30). The drop-out rate will be taken into account in the reporting process, as well as the reasons for exclusion, although this information is free to be provided, as it is contained in the initial information presented to the patient, this information is expanded in the Supplementary Material.

To ensure adequate recruitment of participants to achieve the target sample size, a multidisciplinary approach involving the gynecology, rehabilitation, and physiotherapy departments has been adopted. Collaborators have been provided with information on the study through personal interviews and presentations. Patient recruitment will aim to have socio-demographic diversity that reflects the social background, gender, ethnicity, and educational level of the reference population, while taking into account the specific characteristics of the population.

Prior to the inclusion of patients, the research team will devise the allocation sequence and consecutively assign patients into either the Experimental Group (EG) or the Control Group (CG) through the use of opaque sealed numbered envelopes. This assignment will be done using a computerized random number generator to ensure unbiased allocation. Each participant’s treatment will be administered separately to maintain the confidentiality of study information.

Due to the nature of the intervention in both groups, blinding of patients and physiotherapists will not be feasible. As a result, this study will adopt a single-blind approach, where the evaluator responsible for assessments will remain unaware of the nature of the intervention. Throughout the entire study process, the evaluator will be kept blinded, being unaware of the study objectives and the randomized distribution of patients into study groups. Additionally, access to the randomization sequence will not be provided to the evaluator.

Subjects will undergo an initial evaluation based on clinical parameters, and subsequent follow-up discharge reports will be documented. The collection of data will be performed by the principal investigator and integrated into dedicated research databases.

### 2.4. Intervention

Random allocation will be utilized to assign participants to either the intervention or control groups, which will be achieved by utilizing a random number table. Both groups will receive 3 sessions per week during the 4 weeks of intervention (51).

#### 2.4.1. Control group (CG)

In adherence with clinical practice guidelines, participants assigned to the control group will be provided multidisciplinary rehabilitation programs that involve coordinated delivery of supervised exercise therapy, cognitive-behavioral therapy (including education on pain), as well as therapeutic massage to alleviate LBP during pregnancy. Typical physiotherapy session:

- Control of daily health.
- Analgesic and muscle-relaxing (thermotherapy, tens, therapeutic massage).
- Exercise session:
Initial warm-up: 5–10 min (thoracic, lumbar and pelvic joint mobility exercises adapted to the pregnancy progress).
Strengthening and flexibility exercises (thoracic, lumbar and pelvic joint exercises adapted to the pregnancy progress).
Return to calm: 5 min breath and stretching exercises.
Recording of incidents and patient/physiotherapist feedback.

#### 2.4.2. Experimental group (EG): VR intervention

The experimental group will be treated with the same approach as the control group, as described in the previous section. In addition to the aforementioned treatment, the experimental group will also receive a virtual reality intervention.

The immersive virtual reality (VRi) system is composed by a head mounted display (Oculus Quest, Facebook Inc.) and two controllers. Oculus Quest headset is a wireless and portable Android-based device which supports positional tracking with six degrees of freedom (360°). The internal cameras allow to show an external signal with the user view, which helps to monitoring the patient execution. A Wi-Fi connection and a training area of 2×2 meters are needed.

**Table 2:** Primary and secondary outcomes.

Primary and Secondary Outcomes	Definition	Type
Pain related fear avoidance	FACS	Self-reported
Pain intensity	VAS	Registered / Self-reported
Disability and physical function	RMDQ, ODI	Registered / self-reported
Satisfaction and usability	USEQ	Self-reported

FACS, Fear-Avoidance Component Scale; VAS, Visual Analog Scale; RMDQ, Roland-Morris Disalibity Questionnaire; ODI, Oswestry Disability Index; USEQ, User Satisfaction Evaluation Questionnaire.

After each session, participants will be immersed in a virtual reality landscape provided by the Nature Trek VR software.^2^ Initially, participants will be seated and guided through a five-minute breathing exercise, also known as the “meditation Lotus option.” Subsequently, participants will be encouraged to move freely within a relaxing virtual environment for 15 min, while paying close attention to the calming sounds of nature. The specific themed environment will be selected based on the individual preferences of the participants.

At the start of the research, general care advice, including physical activity and medication intake (the intake of medication shall be permitted, monitored and supervised), is provided to participants. They are also instructed not to engage in any other training programs during the intervention phase. If any participant deviates from the VRi program or experiences any negative incidents, such occurrences are recorded daily. Also, Participants undertaking other training programs during the intervention will be excluded.

### 2.5. Outcomes and instruments

#### 2.5.1. Primary research outcomes

##### 2.5.1.1. Pain related fear avoidance

A new scale called the Fear-Avoidance Components Scale (FACS) was created, which includes important components of previous measures related to fear-avoidance (FA) and additional components of the FA model that were not considered in previous questionnaires. The FACS is based on the most current FA model developed by Vlaeyen (52, 53). The reliability of the FACS was tested, and it demonstrated acceptable test/retest reliability with a correlation coefficient of 0.90–0.94 and high internal consistency (Cronbach α = 0.92) (54). Scale validated in Spanish (54). Pain-related fear avoidance (FA) is a frequently encountered issue among patients who suffer from painful medical conditions and exhibit pain-related catastrophic cognitions, hypervigilance, and avoidance behaviors, which can result in reduced functioning, depression, and disability (55).

##### 2.5.1.2. Pain intensity

The Visual Analog Scale (VAS) has been utilized in earlier research examining alterations in pain, particularly in all randomized trials of treatments for back pain during pregnancy published in or included in the Cochrane and systematic reviews (48). The pain assessment before and after the intervention will be conducted using the visual analog scale (VAS), which consists of a 10-cm scale with 1-cm increments. The participants will be asked to rate their pain on the scale and the score will be recorded. The scale ranges from 0 to 10, where 10 represents the most intense pain. The score indicated by the participants on the scale will be considered as the pain score. Past research has demonstrated that the VAS has a high level of reliability (r = 0.76–0.84) (54). VAS is used in Spanish version and validated in LBP (56, 57).

##### 2.5.1.3. Disability and physical function

In this paper, our focus is on the two back-specific functional measures recommended in the “core-set,” namely the Roland-Morris Disability Questionnaire (RMDQ), in Spanish scale validated (58) and the Oswestry Disability Index (ODI), in Spanish scale validated (59). They are the most commonly used measures of function in back pain research (54).

To measure the severity of disability in participants with less severe LBP, the researchers will use the RMDQ, which consists of 24 categories with yes or no questions. A score of up to 24 can be achieved, with higher scores indicating greater functional disability. The test–retest reliability of the RMDQ has been found to be high, with correlations of 0.91 (same day), 0.88 (1 week), and 0.83 (3 weeks) reported (60, 61).

Participants will complete the Oswestry low-back pain disability index (ODI) to evaluate their functional level during LBP, which consists of 10 questions assessing daily activities. The severity of disability in each category will be scored from zero to five. The validation of the ODI showed high intraclass correlation coefficient (*r* = 0.938) and internal consistency with Cronbach’s alpha of 0.918 (day 1) and 0.895 (day 7) (52, 53).

#### 2.5.2. Secondary research outcomes

##### 2.5.2.1. Satisfaction with virtual reality intervention

The User Satisfaction Evaluation Questionnaire (USEQ) will be used to evaluate participants’ satisfaction with the Virtual Rehabilitation Systems. The USEQ is a questionnaire that measures user satisfaction, a component of usability, in virtual rehabilitation systems. The questionnaire is considered reliable with satisfactory internal consistency (Cronbach alpha coefficient of 0.716), and participants have reported finding it easy to understand with an appropriate number of questions (55). USEQ has been validated in Spanish population (62).

A summary of the variables has been included in Table 2.

### 2.6. Data collection, monitoring and management

**Figure 1:**
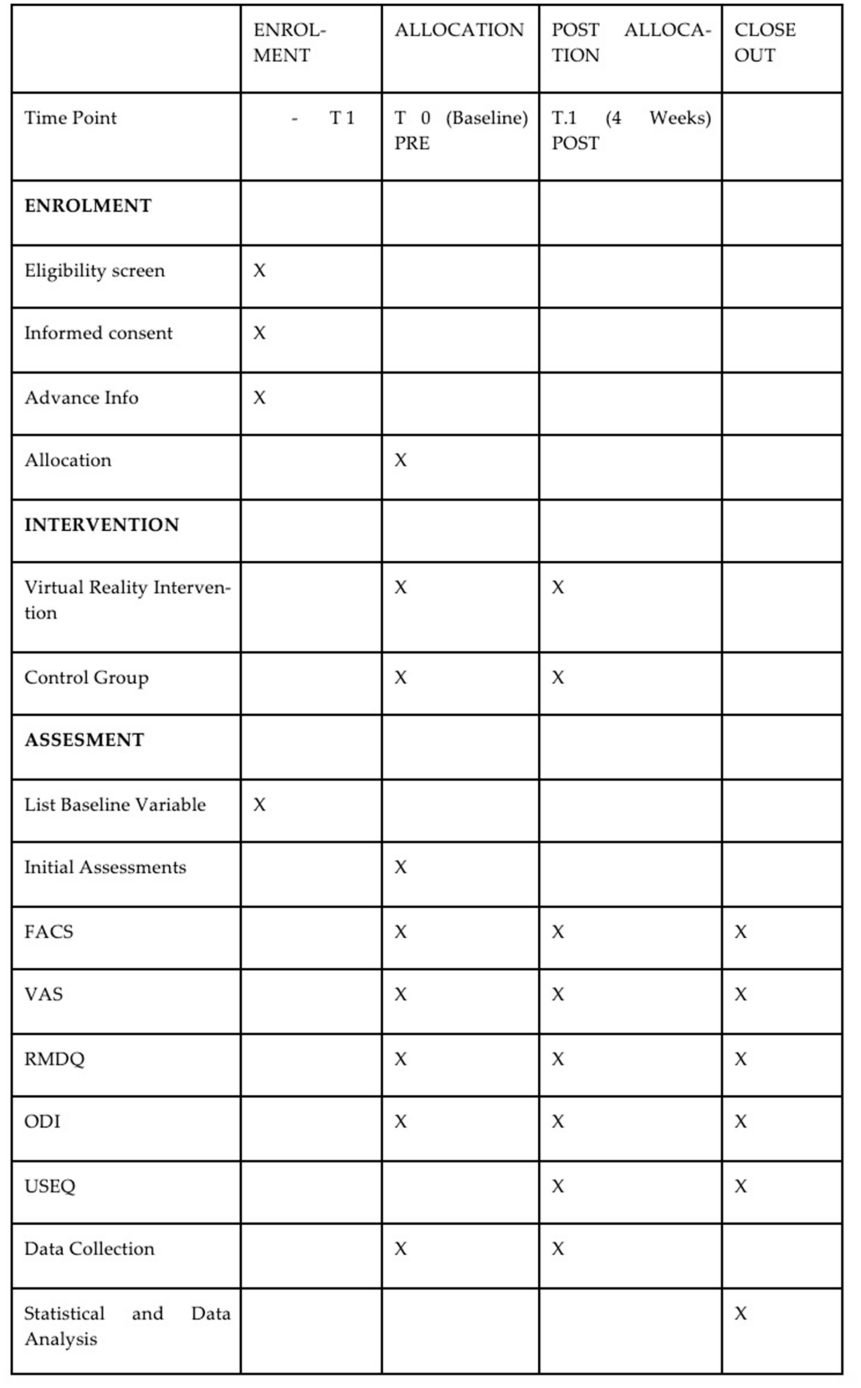
Schedule of enrolment, interventions, and assessments. FACS, Fear-Avoidance Component Scale; VAS, Visual Analog Scale; RMDQ, Roland-Morris Disalibity Questionnaire; ODI, Oswestry Disability Index; USEQ, User Satisfaction Evaluation Questionnaire.

After informing and obtaining consent from participants, the study will collect data by the end of the year 2023, which will be analyzed statistically. The research team, including the rehabilitation and physiotherapy department, will conduct an initial assessment (Pre) and a final 4-week assessment (Post). Schedule of enrollment, interventions, and assessments is shown in Figure 1. The collected data will be aggregated into a research database specifically created for this study, which will be managed by the principal researchers will be conducted using exportable data tables.

**Figure 2:**
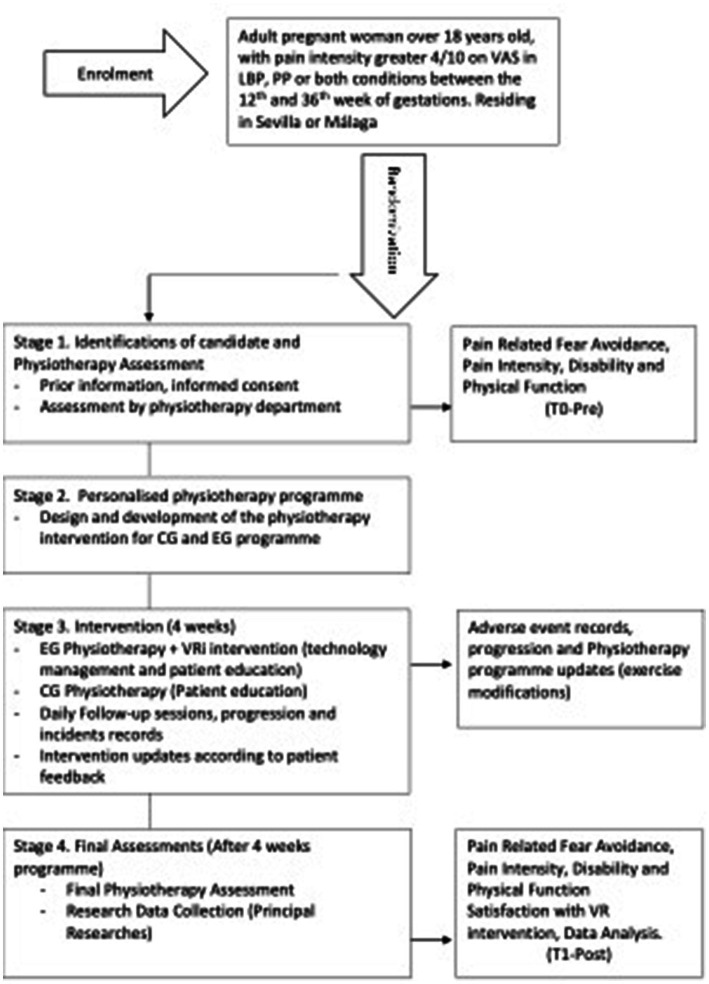
Study design. VAS, Visual Analog Scale; LBP, Low Back Pain; PP, Pelvic Pain: VR, Virtual Reality.

The study has been structured into four stages, which are illustrated in the study design flow diagram depicted in Figure 2:

Stage 1 consists of two processes: the first stage involves identifying potential candidates, providing them with prior information, and obtaining their informed consent to participate. Secondly, the physiotherapy department will conduct assessments, which will include a self-made clinical interview for anamnesis, along with self-administered questionnaires such as FACS, RMDQ, ODI, and 2VAS (T0-Pre). This stage will conclude with a referral to the physiotherapy intervention team.

Stage 2 includes: Design of a personalized physiotherapy program (CG and EG) according with Physiotherapy department plus VRi intervention in the (EG).

Stage 3 includes: participants will receive a 4-week physiotherapy intervention along with a VRi program that is supervised by the physiotherapy department. The intervention will start with a one-on-one session to provide patients with education and training on the use of technology. Daily follow-up sessions will be conducted to monitor the progression of the program and to record any adverse events. Based on the feedback received from the participants, the physiotherapy team will make updates to the program.

Stage 4 includes: the final assessments and evaluation (T1-Post) will be conducted. The physiotherapy team and principal researchers will compile the results of the outcomes after 4 weeks, which will include FACS, RMDQ, ODI, VAS, and USEQ. A satisfaction questionnaire, additionally, it is planned to include the aforementioned data in the research dataset for statistical analysis purposes.

### 2.7. Statistical analysis

The research is a prospective controlled trial with a pre/post design that will be conducted in multiple centers. The results of the trial are intended to be presented in the form of a summary of outcome measures, including estimated effect size and precision. Statistical analysis will be carried out using the “intention to treat” method and for missing data multiple imputation will be used; all data will be collected in a single database and analyzed to evaluate any differences between the randomized groups both for primary outcomes and for secondary outcomes. Patient characteristics will be presented using frequencies and percentages for categorical factors and means and standard deviations for continuous measures to provide comprehensive information for exploration and analysis. Cohen’s d will be used to calculate the effect sizes, which will enable the comparison of results with other studies.

The results will be evaluated by comparing the differences between EG and CG with mixed linear model and T-test statistics to test the hypothesis that the means of two groups are or are not significantly different from each other. The outcome measures will be compared before and after the completion of the 4-week intervention. All statistical analyzes will be carried out using SPSS sofware. Statistical significance will set ap *p* < 0.05 and a unilateral analysis will be made.

## 3. Results

Enrollment first three quarters of 2023. First study results will be reported at the end of the first quarter of the year 2024. The findings from this research will ascertain the viability of implementing a larger intervention on a broader scale. Additionally, this initial study will serve as a pioneering investigation into the impact of the VR intervention on LBP and PP in pregnant women. If the results confirm beneficial effects in the outcomes, this investigation will contribute additional evidence to substantiate the efficacy of utilizing a VR program as a powerful tool in pregnancy with LBP and PP rehabilitation programs. This is the first study that investigates this cause, giving positive results, this study will serve as a basis to extrapolate it to multi-centers, thus being able to carry it out in larger samples, which will allow us to standardize processes.

## 4. Discussion

The results obtained from individual studies propose that certain therapy modalities or a combination of multiple interventions (such as manual therapy, exercise, and education) may be effective to improve pelvic pain and pregnancy related outcomes. However, the current scientific evidence leaves many issues unresolved like type and intensity of exercise and physiotherapy intervention effectiveness for different outcomes. As there is currently no available evidence indicating the superiority of one form of exercise over another, the guidelines suggest exercise programs that take into account individual requirements, inclinations, and capabilities when determining the most appropriate type of exercise. This lack of standardized exercise programs may lead to significant intervention biases in the different studies and consequently the low or moderate level of evidence.

Due to the high prevalence, the recurrence, the interference on daily activities, work capacity and sick leaves, and the increased psychological stress (1–4), LBP is undoubtedly the key clinical sign to address in this population.

The use of immersive virtual reality (VRi) in this case may help alleviate pain by diverting the patient’s attention away from the pain. This is believed to be the psychological effect of being immersed in the virtual space created by VR technology, which can alleviate pain. (63, 64). Additionally, the VR program can create a relaxing atmosphere that may positively affect the patient’s emotional state, thereby reducing their perception of pain (65). There are studies that show how VR can change the patient’s perception due to the focus of attention on the external focus, this approach is very interesting as it can improve their ability to learn (42).

There is evidence that muscle relaxation techniques such as TENS can reduce LBP in pregnant women, however, this is not true of the benefits of yoga for LBP in pregnant women (66, 67). Our approach with Nature Trek VR is to bring relaxation techniques into a virtual environment and test their effects.

Regarding the moment of application of the tear therapy, one of the reasons for putting the relaxation therapy at the end is the ease of use for the physiotherapist as well as for reasons of expectation, as we normally associate the most relaxing techniques at the end of the session. However, there are studies that can be applied during the exercise session itself, which is also appropriate. In both cases, the use is correct, regardless of the moment.

This research aims to gather new information and insights on the practicality of integrating VR programs into clinical environments, with a particular emphasis on discovering new opportunities for interventions that could benefit patients.

However, the use of VR technology may encounter technical challenges such as device malfunctions and technological difficulties. Nevertheless, technical support staff will be available to address these issues. Possible adverse events that may occur include a lack of improvement or positive outcomes for the patient, as well as excessive exercise workload. Among these adverse effects that we may encounter is MS, a pathology that can cause dizziness, vomiting, headache, etc. In particular, we must bear in mind that MS can affect the course of therapy and therefore the success of the treatment. It has been seen that there is a threshold time of onset and that the symptoms may decrease or increase, when the exposure is of slow speed, it may happen that when checked in the Simulator Sickness Questionnaire (SSQ) this is not altered. Therefore, we must take this into account, but we cannot know the degree to which it affects therapeutic success (68, 69). Adverse effects and drop-outs will be taken into account in our case. An important aspect to discuss is the importance of usability and patient satisfaction, i.e., the user experience when using this type of device. We know that this kind of tools can improve adherence, but they also have negative aspects that have to be taken into account such as: cognitive capacity that can interfere directly in usability or simply facts that come from the use of the tool such as motion stinecks. In this case, there are questionnaires to detect this pathology (62). In our study this questionnaire has not been added since the exposure time is short and we do not consider that it can provide us with extra information. Rossettini et al., in their recent reviews, it is shown that patient satisfaction in musculoskeletal pathology is a multidimensional construct influenced by individual patient, clinical and contextual factors. This means that satisfaction can be affected by multifactorial components, not only by the device used (70). Another important aspect to consider is the relationship between the virtual device and its influence as a placebo/nocebo in treatment. This study shows how contextual factors can affect therapeutic success (34). One of the most studied factors is the pain symptom and its relationship to placebo (32, 33). In our case, we might ask ourselves how much influence can the use of virtual devices have on placebo level? If pain is improved, is it really because of the therapy or is it because of the effect? These are questions we do not know how to answer, as future research in our field would be of great interest. Patients will be informed about the importance of reporting any incidents or setbacks in their recovery and their right to withdraw from the research at any time.

Future research directions may involve conducting clinical trials with larger sample sizes and the opportunity to develop a multicenter randomized clinical trial with standardize physiotherapy and exercise programs. The feasibility of this pilot study will serve as a basis for future research in which we would replicate the basic study design, expanding the sample size in different centers, trying to standardize the intervention protocols.

This study protocol represents the first attempt to investigate the impact of VR intervention, combined with physiotherapy, on LBP and PP in a multi-center clinical setting. The effectiveness of this intervention, as well as patient satisfaction, will serve as important indicators of whether this study provides further evidence supporting the use of VR as an effective tool for pregnant women.

### 4.1. Institutional review board statement

This project will adhere to the guidelines outlined in the Declaration of Helsinki (Fortress 2013) and the Standards of Good Clinical Practice. The handling of personal data will comply with Regulation (EU) 2016/679 of the European Parliament and of the Council of 27 April 2016, which pertains to the protection of natural persons regarding the processing of personal data and the free movement of such data, as well as Organic Law 3/2018 of 5 December on the Protection of Personal Data and Guarantee of Digital Rights (71). Only researchers involved in the project will be permitted to access the research data. Each subject’s information will be linked with a unique numerical identification code and will be the sole means of identifying the patient for the purposes of data processing and analysis. This trial has been approved by the Andalucía Ethics Committee with HIP version 1928-N-21. It has also been registered in the clinicaltrials.gov database under the trial registration number NCT05571358.

### 4.2. Informed consent statement

All subjects participating in the study provided informed consent prior to their inclusion. To do so, participants were asked to read and sign the patient information sheet and consent form. They were also informed of their right to revoke their consent at any time without having to provide a reason and without any adverse consequences.

## Dissemination

The results of this study will be published in academic journals and presented in both the academic and public domain, including at scientific conferences and in the media in public engagement forums. Patient confidentiality will be maintained in all of the above.

## Ethics statement

The studies involving human participants were reviewed and approved by the institutional ethics committee of Andalucía with internal code 1928-N-21. Participants gave written informed consent before enrolling in the study.

## Author contributions

F-JG-L, J-MP-B, NM-M, M-JE-P, AL-G, and RM-V have played important roles in the development of this article. Specifically, F-JG-L, J-MP-B, and NM-M coordinated the project, contributed to the conception and design of the study, and were involved in writing the manuscript. M-JE-P and RM-V provided methodological guidance. F-JG-L and AL-G were responsible for coordinating the intervention protocols, patient recruitment, and reviewing the manuscript for spelling and grammar. All authors contributed to the article and approved the submitted version.

## Conflict of interest

The authors declare that the research was conducted in the absence of any commercial or financial relationships that could be construed as a potential conflict of interest.

## Publisher’s note

All claims expressed in this article are solely those of the authors and do not necessarily represent those of their affiliated organizations, or those of the publisher, the editors and the reviewers. Any product that may be evaluated in this article, or claim that may be made by its manufacturer, is not guaranteed or endorsed by the publisher.

## Figures and Tables

**Table:** 

LBP	Low Back Pain
PP	Pelvic Pain
VR	Virtual Reality
TENS	Transcutaneous electrircal nerve stimulation
SQUIRE	Standards for Quality Improvement and Excellence in Reporting
CONSORT	Consolidated Standards of Reporting Trials
VAS	Visual Analog Scale
CG	Control Group
EG	Experimental Group
VRi	Immersive Virtual Reality
FACS	The Fear-Avoidance Components Scale
ODI	Oswestry Disability Index
RMDQ	Roland-Morris disability Questionnaire
USEQ	User Satisfaction Evaluation Questionnaire
MS	Motion Sickness
SSQ	Simulator Sickness Questionnaire

## Footnotes

^1^https://www.ai-therapy.com/psychology-statistics/sample-size-calculator

^2^https://naturetreksvr.com/

## Supplementary material

SUPPLEMENTARY Data Sheet S1 Minimal sample size.

SUPPLEMENTARY Data Sheet S2 Informed consent.

SUPPLEMENTARY Data Sheet S3 Prior information for the patient.

SUPPLEMENTARY Data Sheet S4 SPIRIT 2013 checklist.
