# Supplementary figures and images for: Virtual reality to improve low-back pain and pelvic pain during pregnancy: a pilot RCT for a multicenter randomized controlled trial
Source: Front Med (Lausanne). 2023 Sep 4;10:1206799. doi: 10.3389/fmed.2023.1206799 (PMC10507341; doi:10.3389/fmed.2023.1206799)

1. **MINIMAL SAMPLE SIZE**


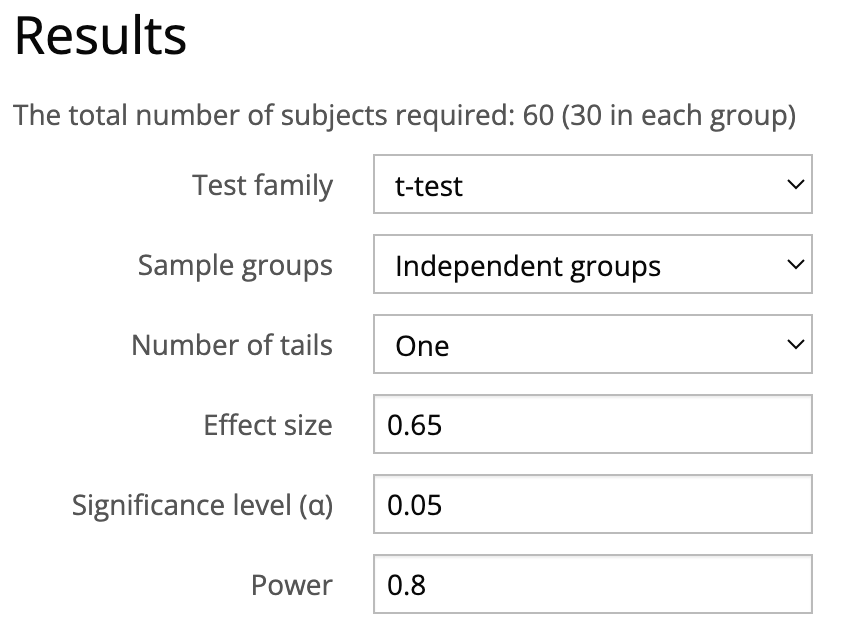

Supplement: SUPPLEMENTARY Data Sheet S1 — Minimal sample size. [file Data_Sheet_1.docx]
