# Supplementary material for: Virtual reality to improve low-back pain and pelvic pain during pregnancy: a pilot RCT for a multicenter randomized controlled trial
Source: Front Med (Lausanne). 2023 Sep 4;10:1206799. doi: 10.3389/fmed.2023.1206799 (PMC10507341; doi:10.3389/fmed.2023.1206799)
Supplement: SUPPLEMENTARY Data Sheet S2 — Informed consent. [file Data_Sheet_2.docx]

2. INFORMED CONSENT

Informed Consent - Written Consent of the Patient

I (Name and Surname): ...........................................................................................

1. I declare that I have read the Patient Information Sheet that accompanies this consent.

2. I have been able to ask questions about the study. All questions were answered to my satisfaction.

3. I have spoken to the reporting healthcare professional:.....................................

4. I understand that my participation is voluntary and I am free to participate or not in the study.

5. I have been informed that all data obtained in this study will be confidential and will be treated in accordance with the Organic Law on Personal Data Protection 3/2018.

6. I understand that I can withdraw from the study:

- Whenever I want

- Without having to give explanations

- Without affecting my medical care.

I freely give my agreement to participate in the project entitled

I GIVE

I DO NOT GIVE

Signature of Patient Signature of reporting health professional

First and Last Name:………………. First and Last Name:………..

Date: ……………………………… Date:………………………
