# Supplementary material for: Virtual reality to improve low-back pain and pelvic pain during pregnancy: a pilot RCT for a multicenter randomized controlled trial
Source: Front Med (Lausanne). 2023 Sep 4;10:1206799. doi: 10.3389/fmed.2023.1206799 (PMC10507341; doi:10.3389/fmed.2023.1206799)
Supplement: SUPPLEMENTARY Data Sheet S3 — Prior information for the patient. [file Data_Sheet_3.docx]

3.PRIOR INFORMATION FOR THE PATIENT

**TITLE:** "Virtual reality to improve low back and pelvic pain during pregnancy: A Pilot RCT for a multicenter randomized controlled trial".

Department of Physiotherapy, Faculty of Health Sciences, University of Malaga. Principal Researcher: Francisco José García López, fjgarlop@uma.es.

**WHY ME?**

Because you are over 18 years old and have recently been selected by _ _ _ _ _ _ _ _ _ _ _ _ _ _ _ _ _ _ _ _ _ _ _ _ _ _ _ _ _ _ _ _ _ _ _. Therefore, the research team has considered that you can benefit from the means offered by this research project.

**WHAT DOES THE PROJECT CONSIST OF?**

An intervention accepted and validated by the scientific community is applied. Similar studies have shown that it can be a beneficial tool and that it can have positive effects or in any case never be detrimental to your condition. We want to test its effectiveness in certain patients, as well as to know if its implementation is satisfactory.

**WHAT HAPPENS IF I DECIDE TO APPLY OTHER MEASURES IN ADDITION?**

Your consultant will inform you during the research period what kind of medical care you will receive and how you should report any other type of intervention that may influence the results.

**WHAT HAPPENS AFTER THE INTERVENTION WEEKS?**

Your counsellor during the research period will inform you of the recommendations to follow after the intervention is over.

**IF I DON'T FEEL LIKE IT, I CAN'T, I DON'T WANT TO CONTINUE IN THE STUDY, WHAT HAPPENS?**

You can decide at any time to be included or excluded from the project without giving any explanation if you wish to do so. When withdrawing from treatment or intervention, you will be asked for the reasons for withdrawal, which you are not obliged to answer.

**WHAT GUARANTEES DO I HAVE IF I DECIDE TO PARTICIPATE?**

In addition, the monitoring will be exhaustive by researchers with more than 20 years of professional experience.

**Basic information on data protection**

In accordance with the provisions of Regulation (EU) 2016/679 of the European Parliament and of the Council, and the Organic Law on Personal Data Protection 3/2018 and Guarantee of Digital Rights, we inform you that the personal data provided will be processed exclusively by the Principal Investigator and the members of the research team.

Rights: You have the right to access, rectify and delete the data, as well as other rights that can be exercised by contacting fjgarlop@uma.es.
